# Supplementary material for: Laser Ablation on Isostatic Graphite—A New Way to Create Exfoliated Graphite
Source: Materials (Basel). 2022 Aug 9;15(16):5474. doi: 10.3390/ma15165474 (PMC9410218; doi:10.3390/ma15165474)
Supplement: Supplementary file 1 [file materials-15-05474-s001.zip › materials-1808322-supplementary.pdf]

Supplementary Information

Surface morphology changes in graphite by high-peak-power nanosecond-pulse laser ablation: An effort to create nano-carbon with graphene-like surface topology

Authors: Maria Isabel Sierra Trillo[1,2], Yi Thomann[*1], Ralf Thomann[1,3], Ingo Krossing[1,2], Ralf Hanselmann[3], Rolf Mülhaupt[1,3]

[1]*Freiburger Materialforschungszentrum, Stefan-Meier-Straße 21, D-79104 Freiburg i. Br.,*

[2]*Institut für Anorganische und Analytische Chemie, Albert Str 21, D 79104 Freiburg i. Br.,*

[3]*Institut für Makromolekulare Chemie, Stefan-Meier-Straße 31, D 79104 Freiburg i. Br.,*

Albert-Ludwigs-Universität Freiburg

[*]*Corresponding author: Yi.thomann@fmf.uni-freiburg.de*

Material: isostatic graphite

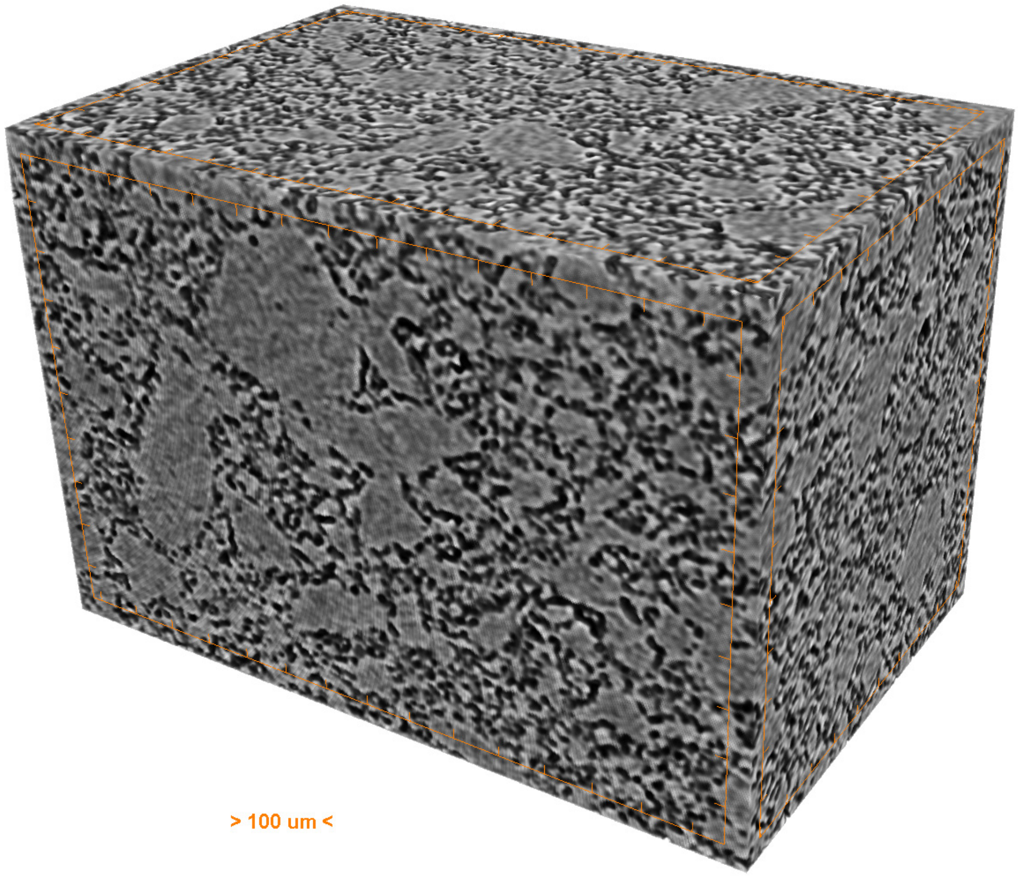

Figure S1: 3D porous structure of the used isostatic graphite, characterized by a μ-CT (SkyScan 1272). The black color represents the pores and gray color the graphite particles.

Prove of liquid carbons

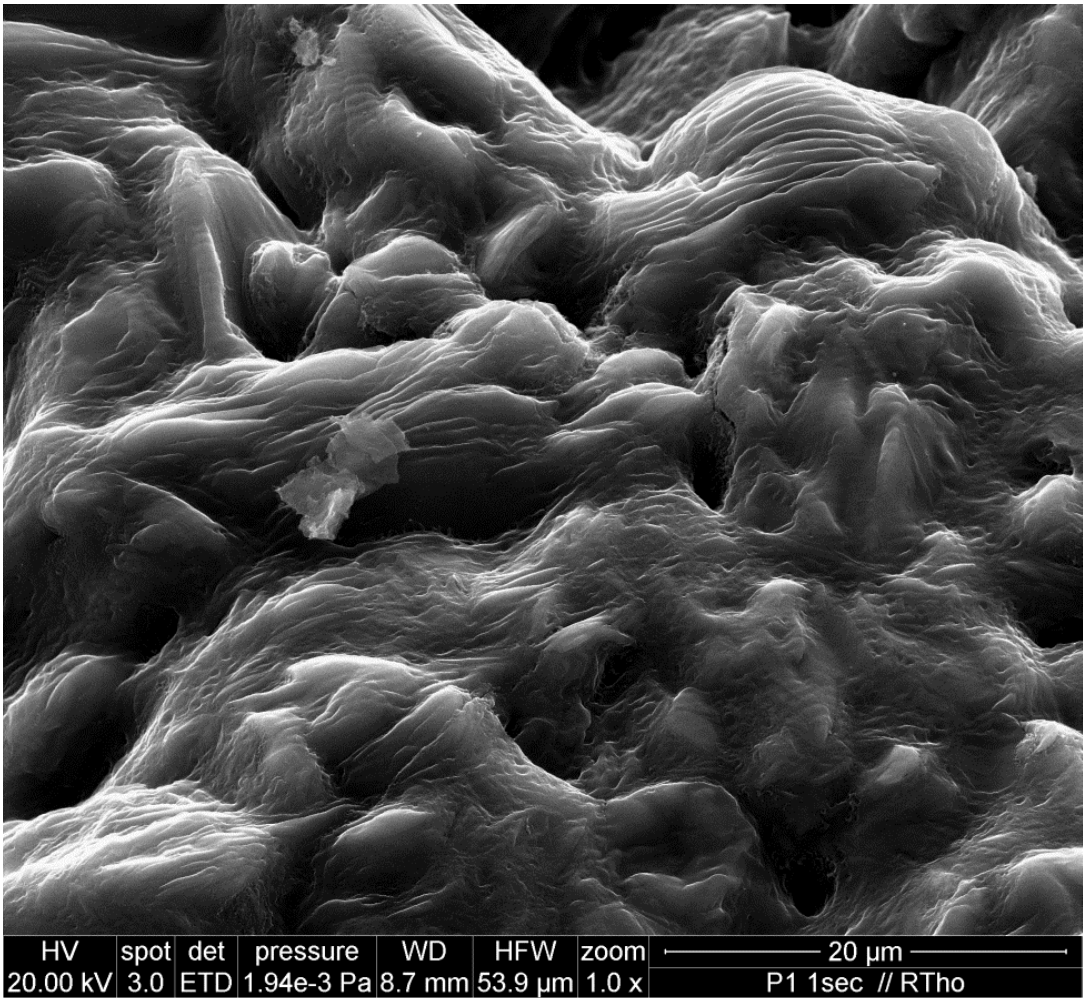

Figure S2a: Whirling like structure in zone 1, indicating the liquid state of carbon; 3 mJ pulse energy and 1 second irradiation time; from pristine graphite; zone 1

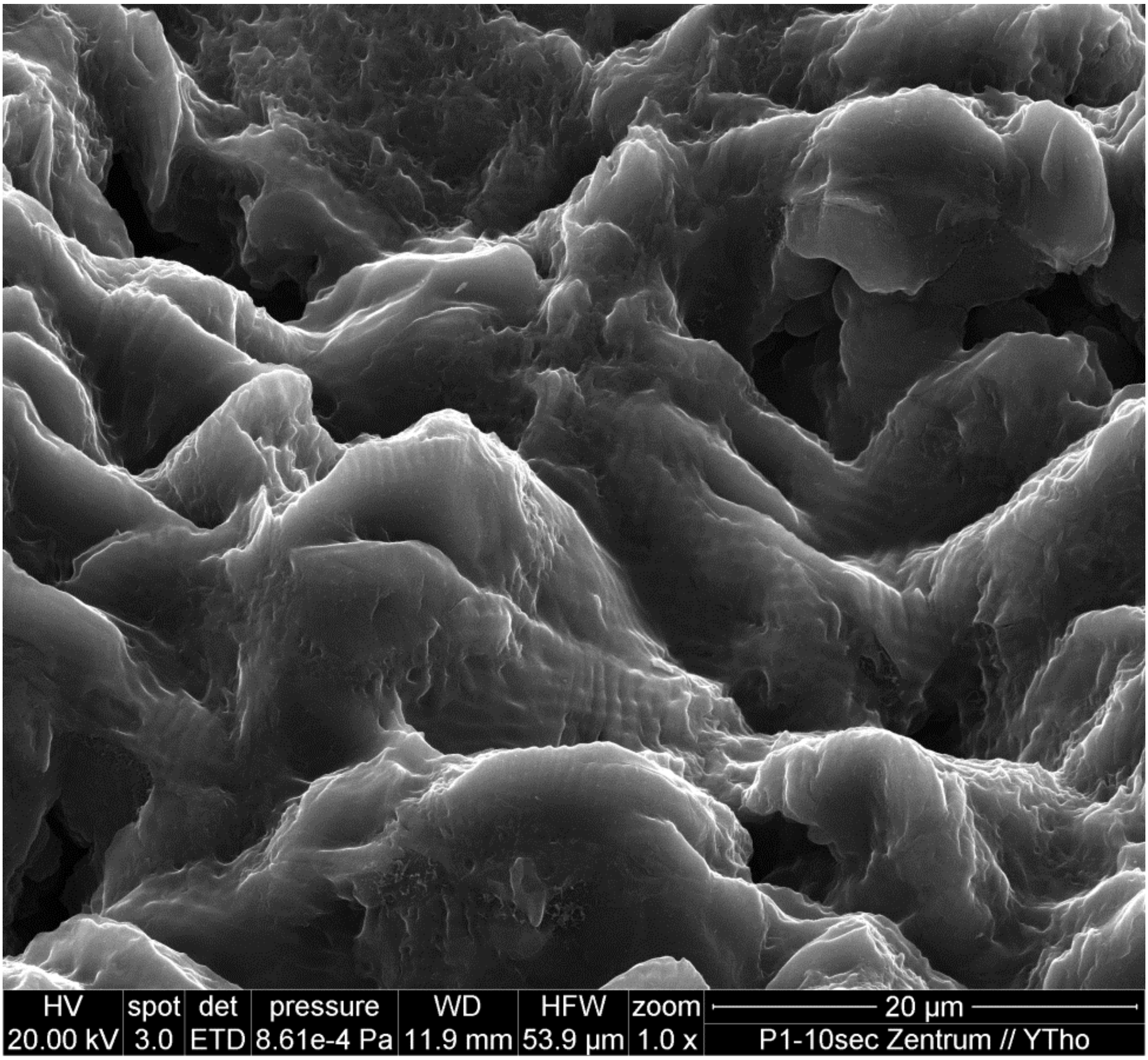

Figure S2b: SEM image center of a crater, showing whirling like structure and some interference pattern; 3 mJ laser pulse energy and 10 seconds irradiation time; from pristine graphite; from pristine graphite; zone 1.

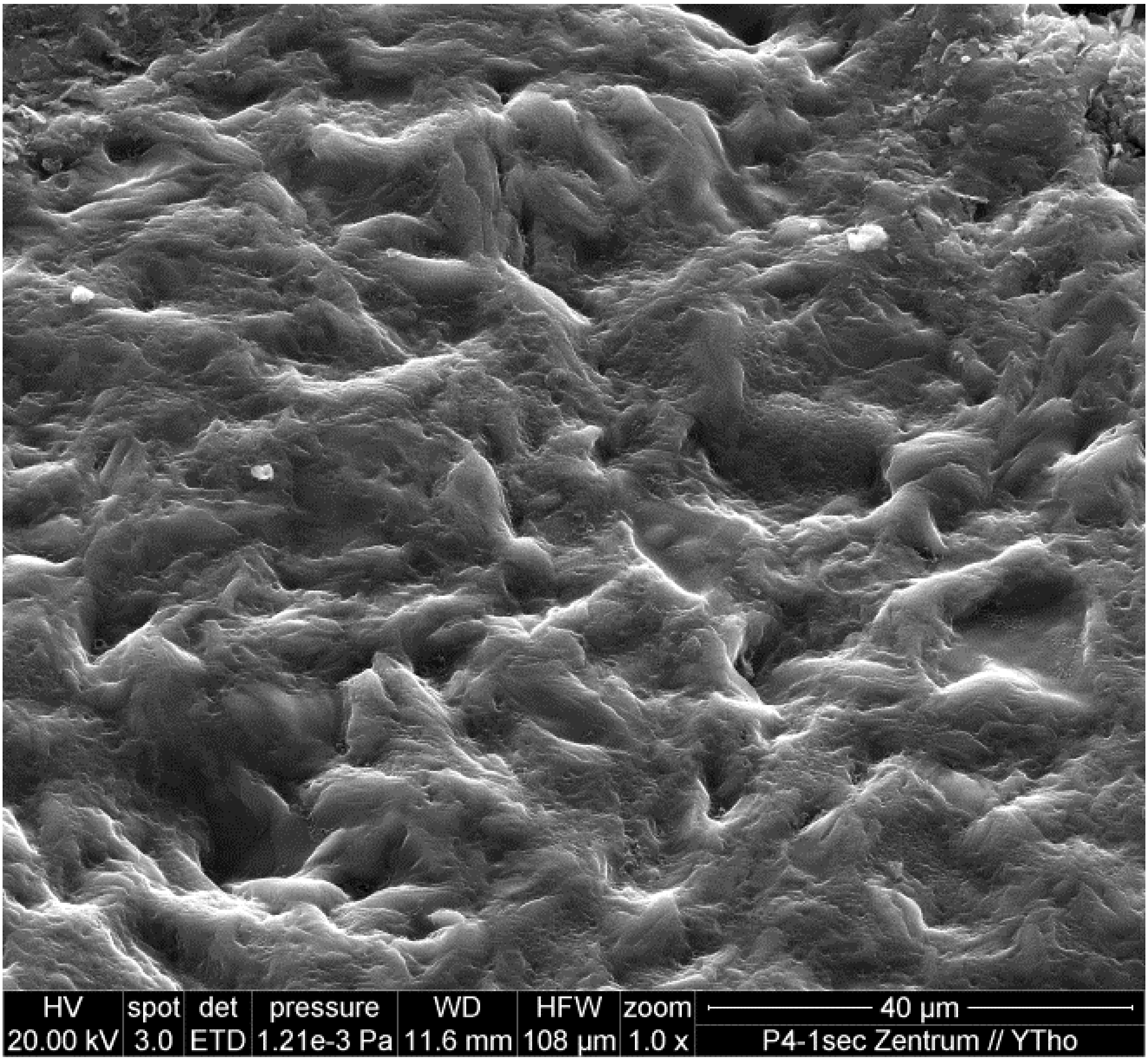

Figure S2c: Whirling like structure in zone 1, indicating the liquid state of carbon; 24 mJ pulse energy and 1 second irradiation time; from pristine graphite; zone 1

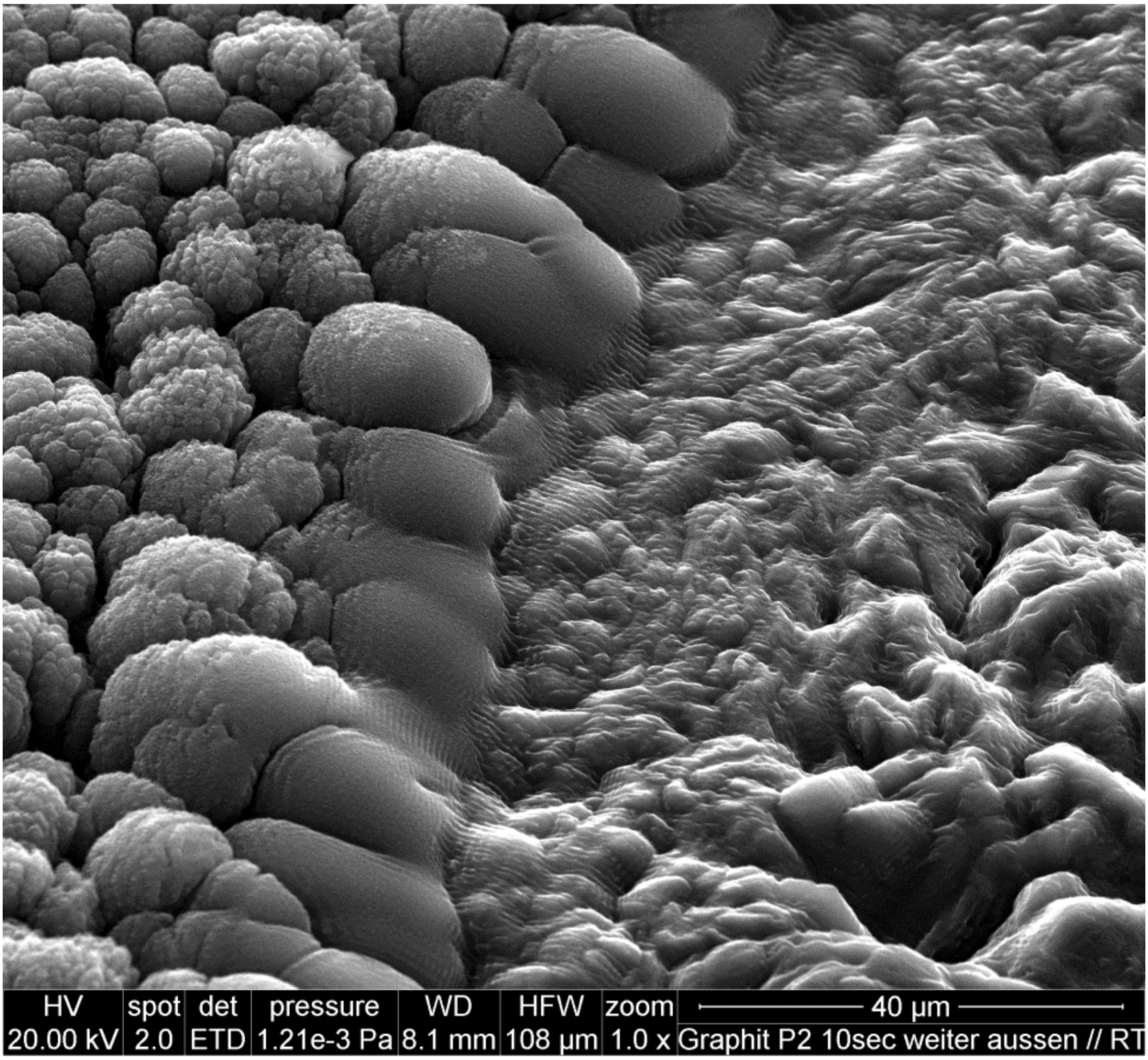

Figure S3a: 9 mJ, 10 seconds irradiation time; from pristine graphite; from zone 1 (right) to zone 3 (left)

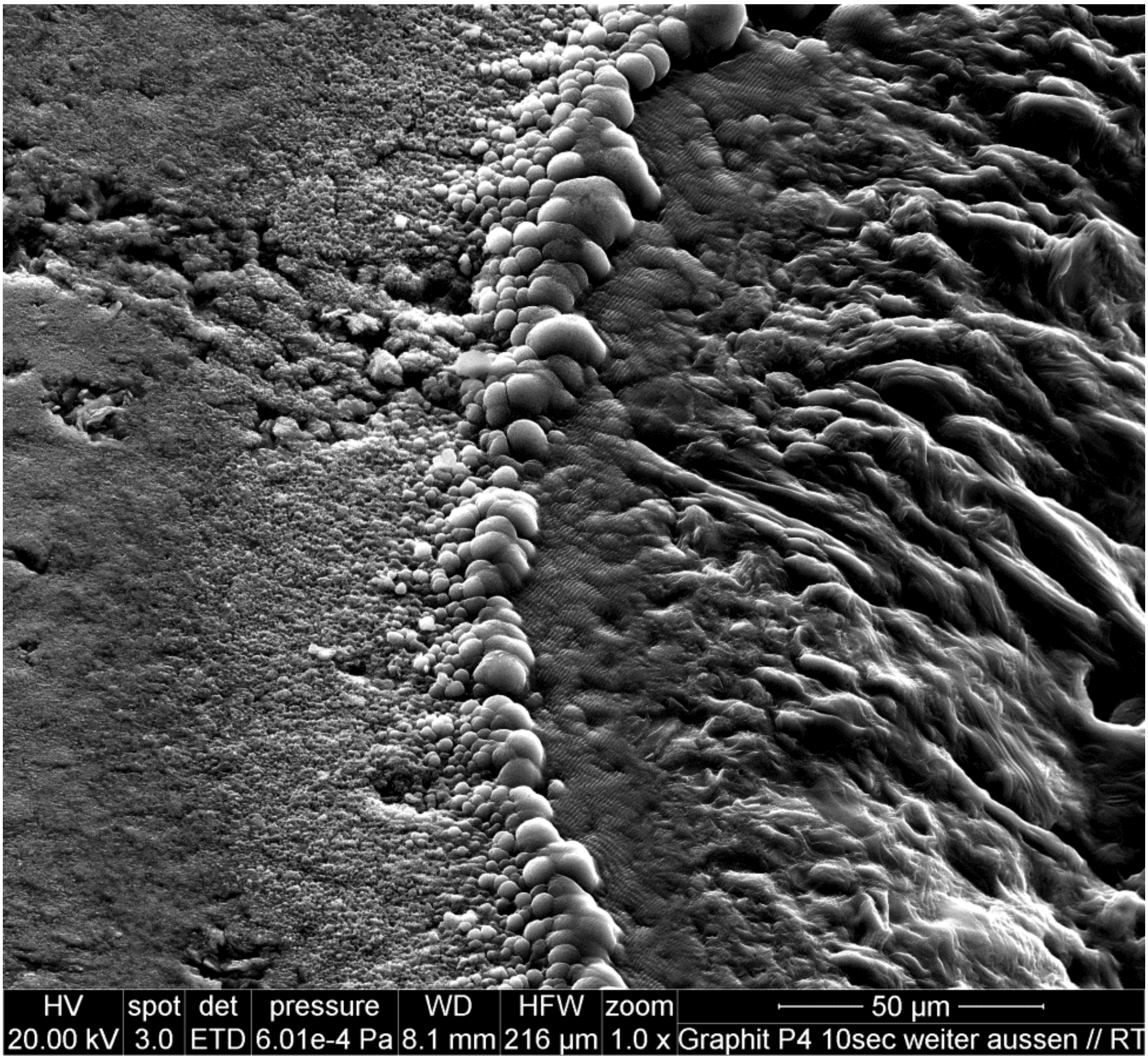

Figure S3b: 24 mJ, 10 seconds irradiation time; pristine graphite; from zone 1 (right) to zone 4 (left)

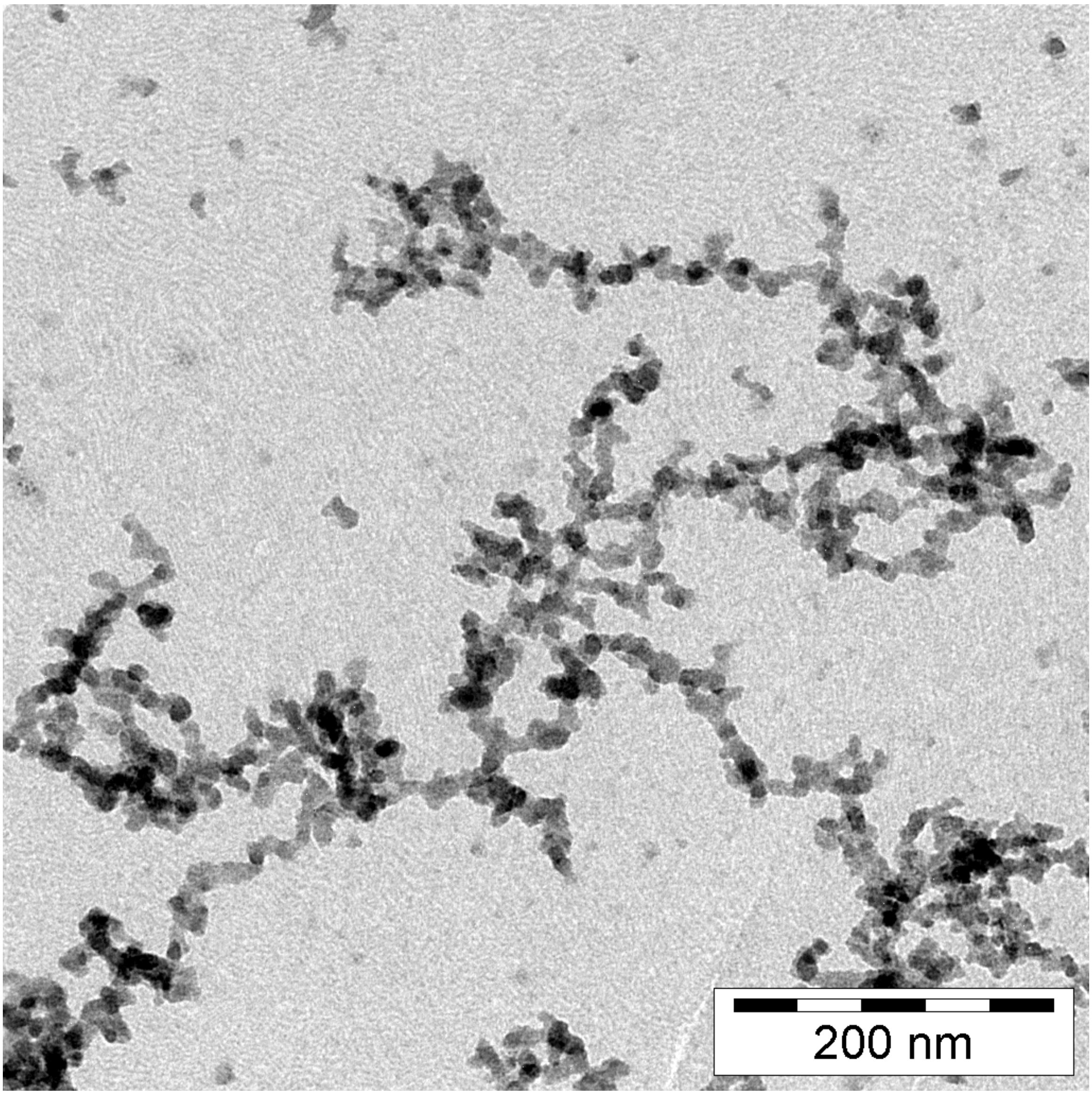

Figure S4 TEM image of the Carbon Fragments gathered on a TEM-Carbon-grid from the Zone 4 of a graphite target ablated

Confocal micro Raman measurements:

The ablated surface, especially the zone 3, were analyzed using a confocal micro-Raman spectrometer LabRam from Horiba Jobin Yvon GmbH , employing an excitation laser with a wavelength of 632.819 nm, and the spectra were acquired with a 100X objective at a laser power of 0.1 mW.

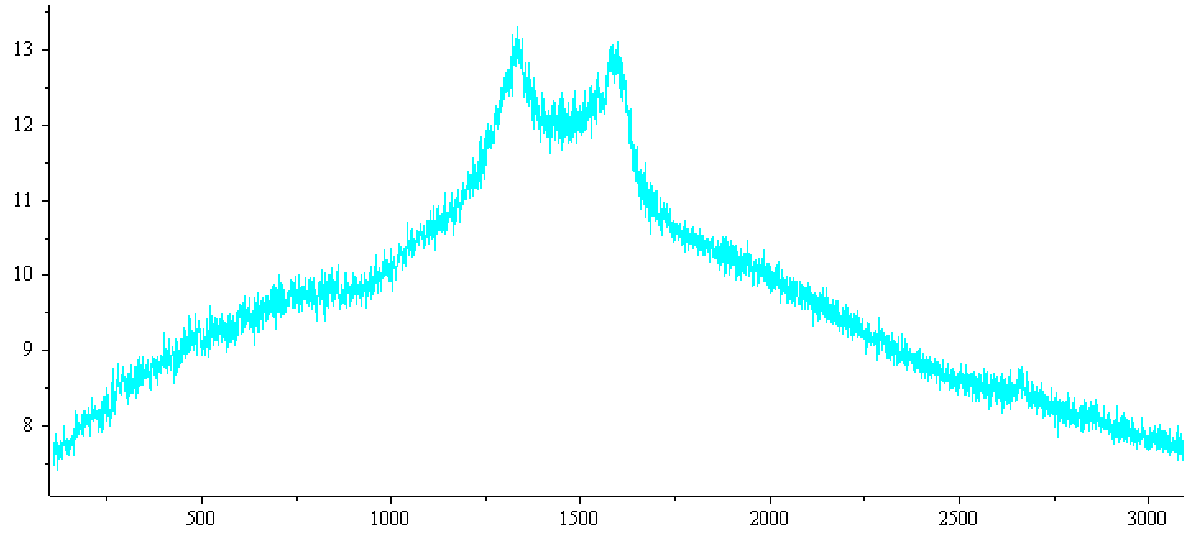

Figure S5a: Raman spectrum, zone 3, irradiation time 10 seconds with 16 mJ pulse energy; on pristine graphite. The signals of graphite peaks are quite weak in comparison to none irradiated graphite. The big halo between 1000 and 2000 cm-1 indicates, most of the materials in zone 3 are amorphous carbon or of some thermally decomposed graphite. A confocal raman microscope of the Yvo type, with a laser beam size 2 µm was used.

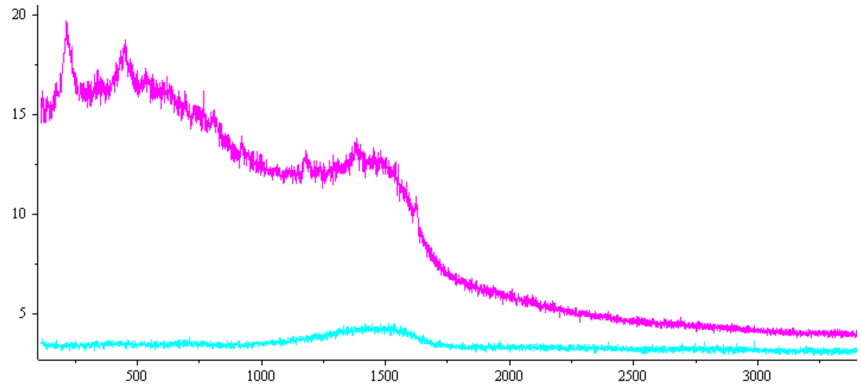

Figure S5b: Raman spectrum of deposits gathered onto a TEM-carbon-grid (red) and of the carbon-grid (blue). Some decomposition product from fullerene like materials could be supposed, which is in accordance to the MALDI-TOP results. Zone 4. Correspond to the TEM image in Figure S4

MALDI-TOF investigation

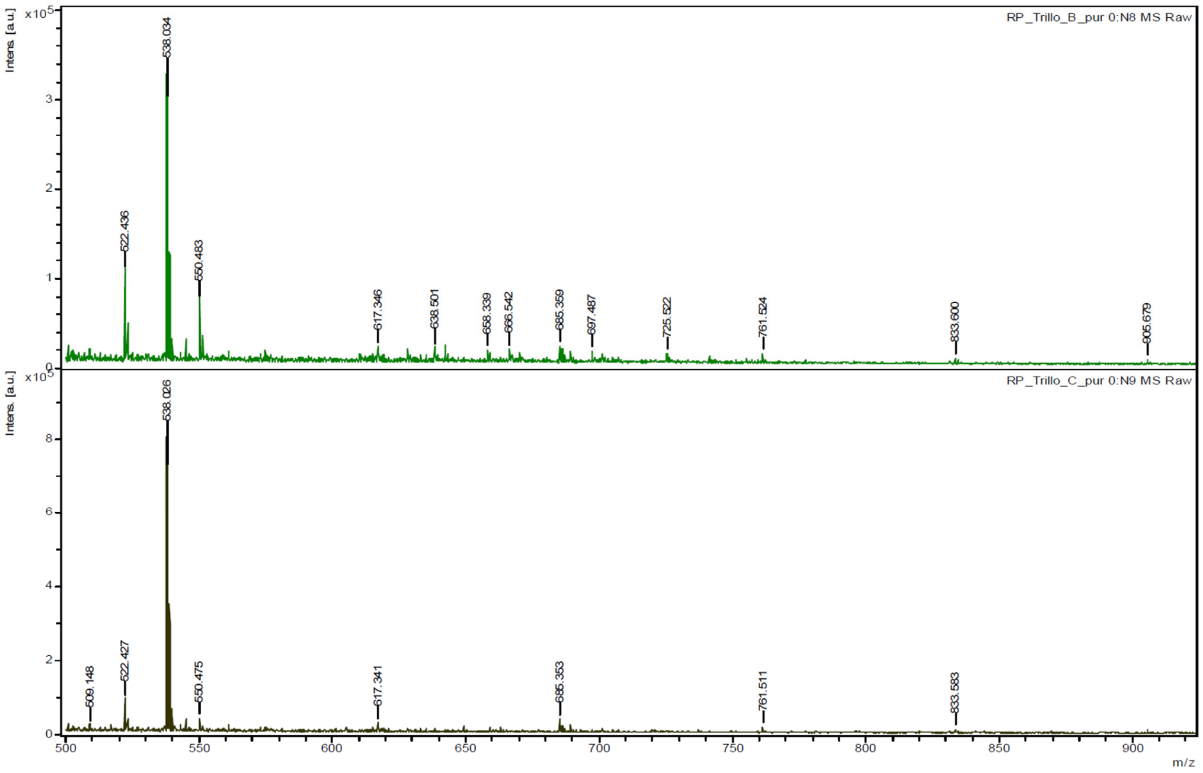

Figure S6: MALDI-TOF spectra: deposited carbon fragments found in zone 4; pristine graphite, 16 mJ (above) and 24 mJ (below) pulse laser and 3 minutes irradiation time; Ablation without presence of a liquid medium.

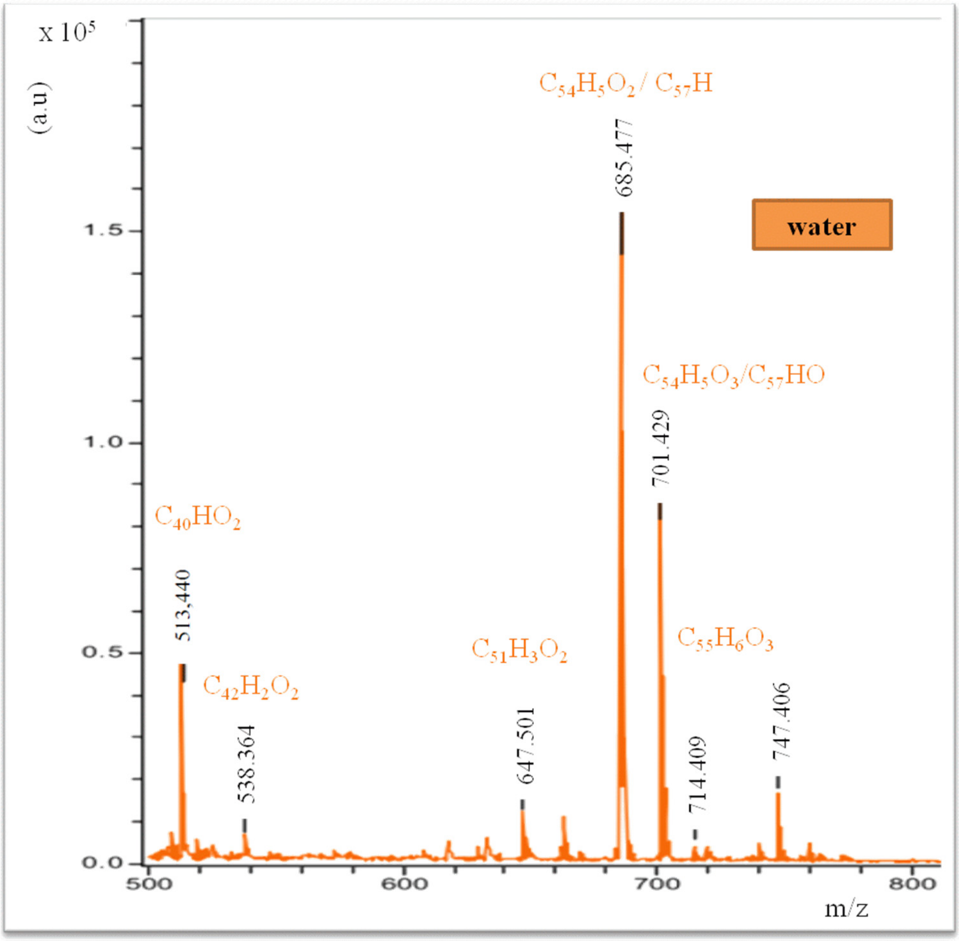

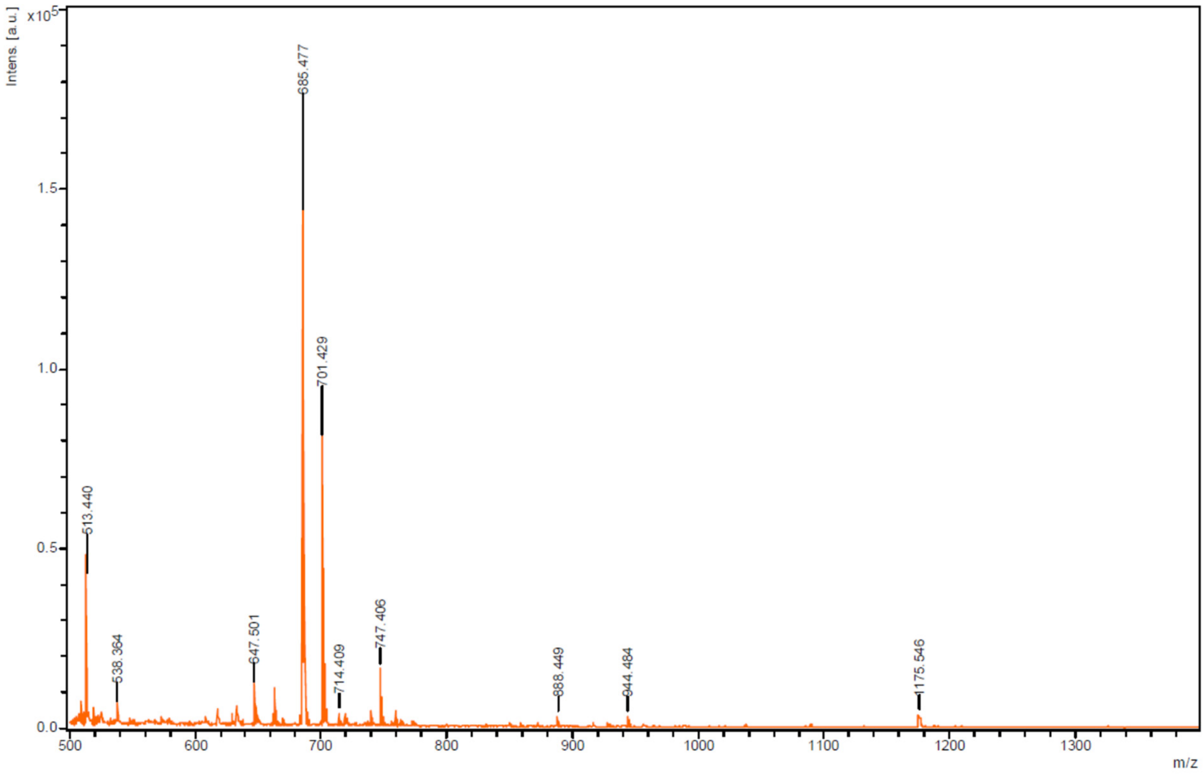

Figure S7: *MALDI-TOF spectra of the water after laser irradiation. Ablation with liquid medium water.*

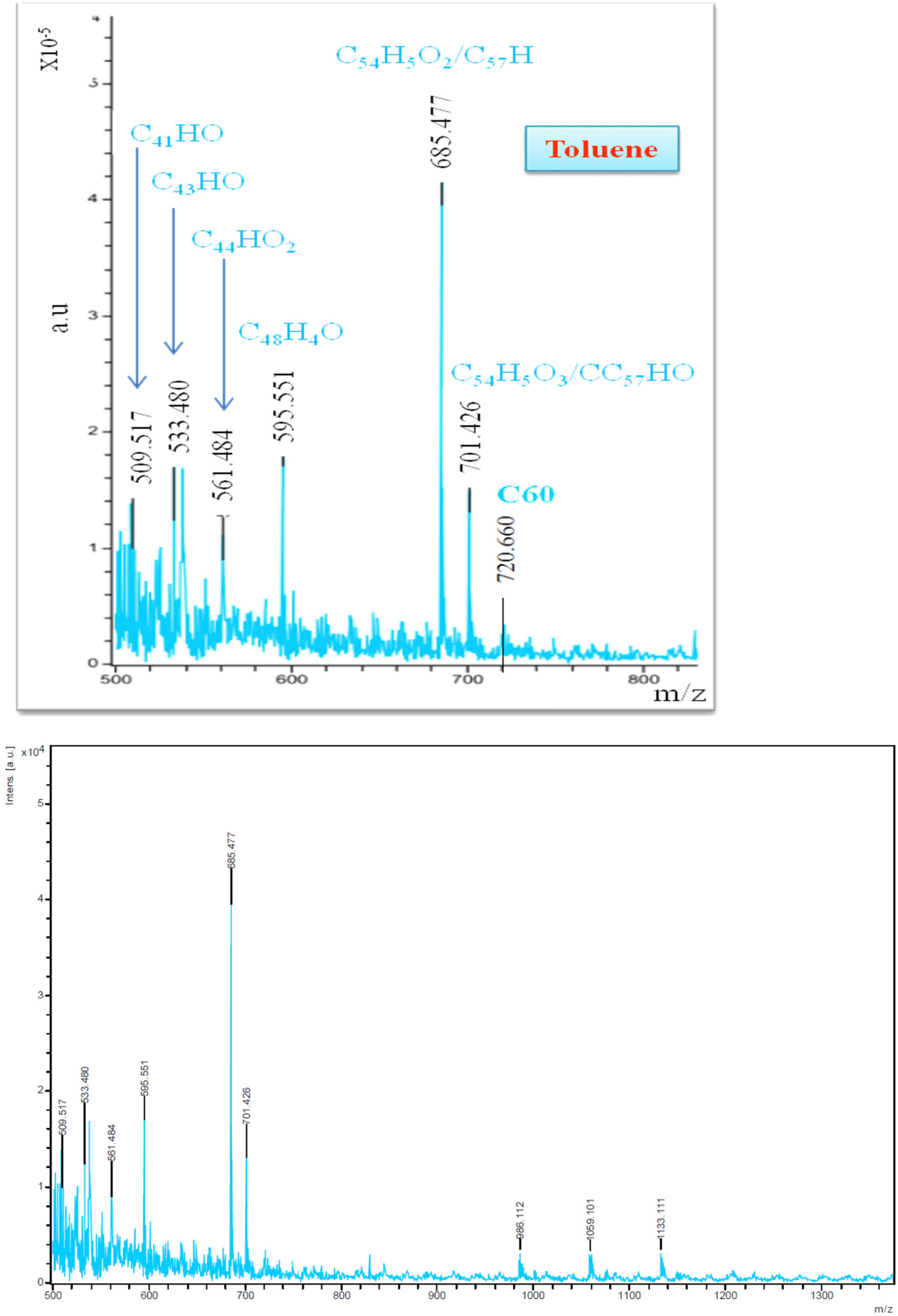

Figure S8: *MALDI-TOF spectra of toluene after laser irradiation; Ablation with liquid medium toluene.*

Proposed chemical composition

3mJ laser energy and a 7 second irradiation time bromine intercalated graphite

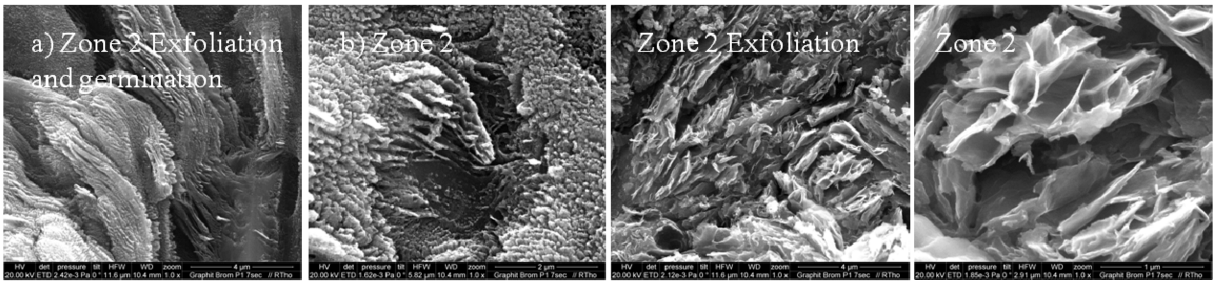

Figure S9: SEM images of the different areas of zone 2 obtained when the graphite target was intercalated with bromine. Bromine treated graphite. Zone 2.

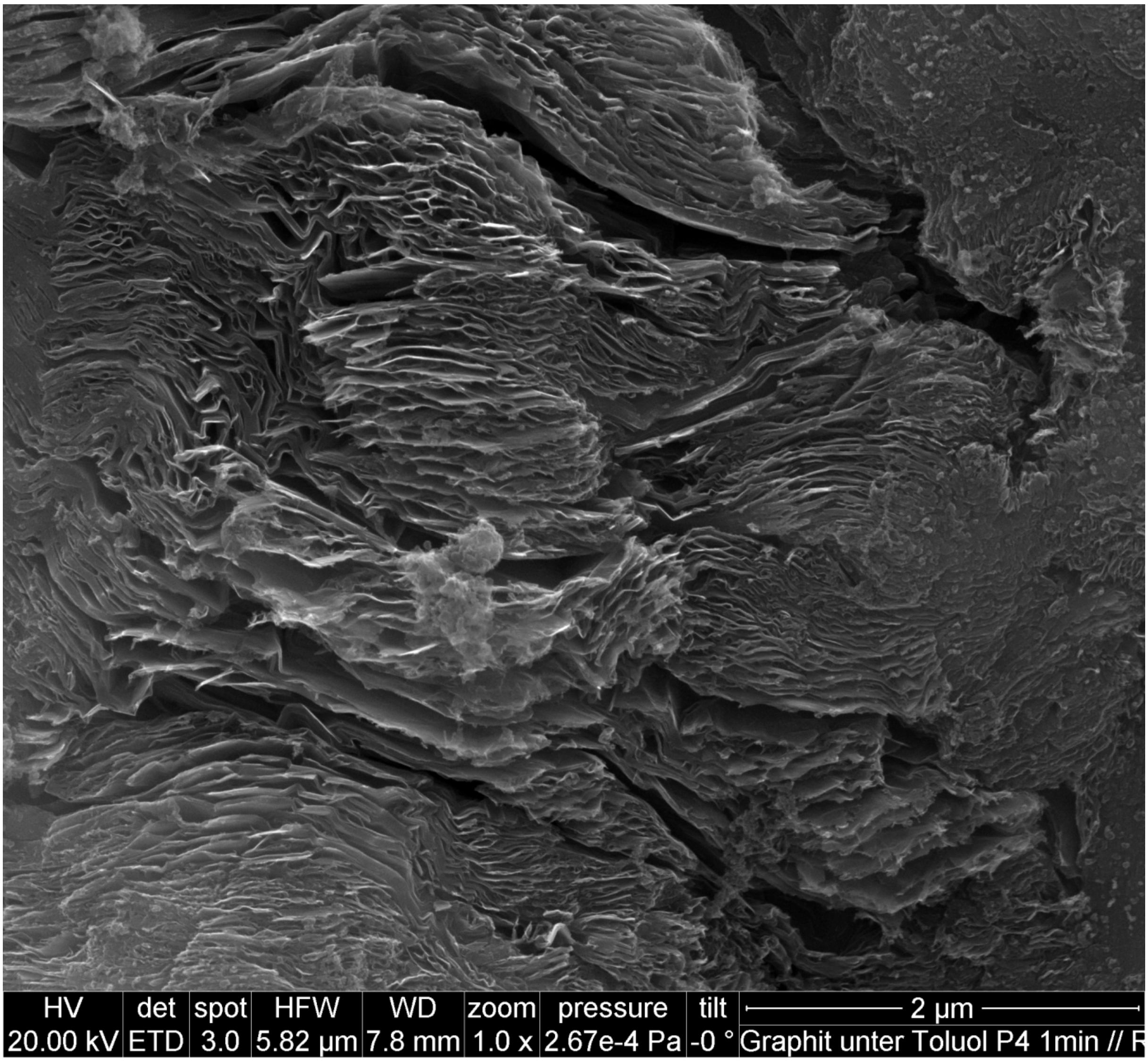

Figure S10: High magnification SEM image of the exfoliated graphite structure generated by ablation under toluene liquid medium; zone 3; 24 mJ pulse energy and 1 minute irradiation time.

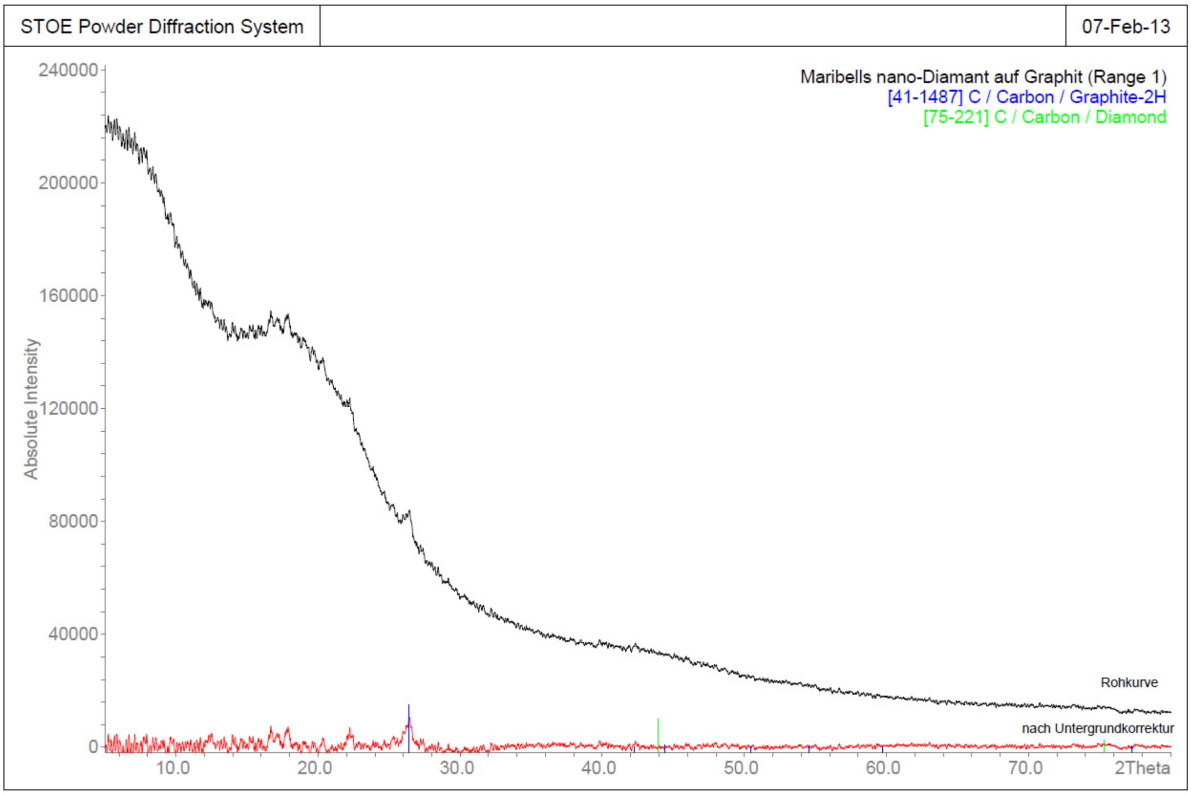

Figure S11: XRD of the sample ablated graphite was peeled by a Tesa-film and the carbon sticking to the film was measured by XRD. Dry ablated with 24 mJ energy and 1 min irradiation time.

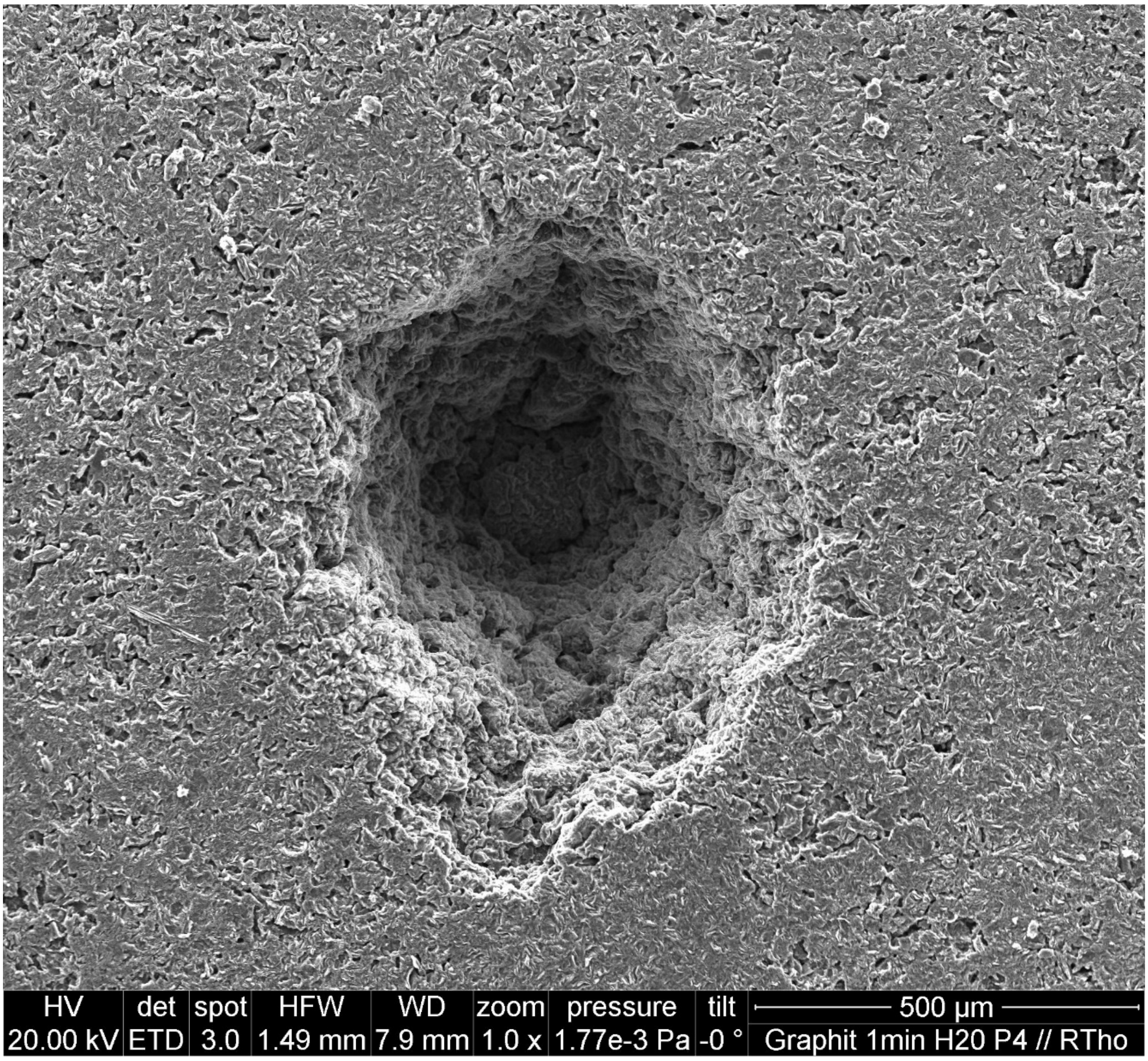

Figure S12: Low magnification SEM image of the crater generated by ablation under water liquid medium; zone 3; 24 mJ pulse energy and 1-minute irradiation time.
